# Screening of liquid media and fermentation of an endophytic *Beauveria bassiana* strain in a bioreactor

**DOI:** 10.1186/s13568-014-0047-6

**Published:** 2014-05-29

**Authors:** Rieke Lohse, Desiree Jakobs-Schönwandt, Anant V Patel

**Affiliations:** 1 Department of Engineering and Mathematics, University of Applied Sciences, Wilhelm-Bertelsmann-Str. 10, Bielefeld 33602, Germany

**Keywords:** Submerged culture, Fermentation, Beauveria bassiana, Endophyte, Blastospores, Submerged conidiospores, Biological control

## Abstract

A novel approach for biological control of insect pests could be the use of the endophytic entomopathogenic *Beauveria bassiana* isolate ATP-02. For the utilization of the endophyte as a commercial biocontrol agent, the fungus has to be mass-produced. *B. bassiana* was raised in shake flask cultures to produce high concentrations of total spores (TS), which include blastospores (BS) and submerged conidiospores (SCS). The highest concentration of 1.33×10^9^ TS/mL and the highest yield of 5.32×10^10^ TS/g sucrose was obtained in the TKI broth with 5% sugar beet molasses which consists of 50% sucrose as a carbon source. In spite of the lower sugar concentration (2.5%) the amount of TS could be increased up to 11-times in contrast to the cultivation with 5% sucrose. The scale-up to a 2 L stirred tank reactor was carried out at 25°C, 200–600 rpm and 1 vvm at pH 5.5. A TS yield of 5.2×10^10^ TS/g sucrose corresponding to a SCS yield of 0.2×10^10^ SCS/g sucrose was obtained after 216 h. With regards to the culture medium the cost of 10^12^ TS amounts to 0.24 €. *Plutella xylostella* larvae, which were fed with oilseed rape leaves treated with spores from fermentation resulted in 77 ± 5% mortality. Moreover, spores from submerged cultivation were able to colonize oilseed rape leaves via leaf application. This is the first report of fermentation of an endophytic *B. bassiana* strain in a low-cost culture medium to very high yields of TS.

## Introduction

In the past decades, many microorganisms have been isolated and investigated for use as a biocontrol agent. Now, many promising strains are available for release into the environment and especially with the renewed interest in biocontrol await further exploitation for large-scale application in agriculture (Glare et al. [2012]). The first step for commercialization of a biocontrol agent like *Beauveria bassiana* is the mass-production by fermentation (Burges [1998]; Ravensberg [2011]). *B. bassiana* strains that were applied to the insect and act on the outer surface of the plant show efficacy against a wide range of insect pests and have the potential of becoming a cost-effective biocontrol agent (Khachatourians [1986]). However, the approved products of *B. bassiana* contain aerial conidia (AC), which are produced by either a solid-state or a diphasic fermentation. These processes are in classical biotechnology considered to be labour-intensive and unsuitable for conventional production of fungal biomass (Feng et al. [1994]; Patel et al. [2011]; Ravensberg [2011]; Rombach et al. [1988]). In contrast to these propagules, blastospores (BS) and submerged conidiospores (SCS) would be produced in submerged cultivations in a shorter time with higher yields and state-of-the-art process control. Furthermore, it was shown that BS and SCS of a *B. bassiana* isolate are as virulent to grasshoppers as the AC (Hegedus et al. [1992]). Until today, no products with BS or SCS of *B. bassiana* are available. A few reports on growth requirements and shake flask culture of *B. bassiana* strains show the best growth and germination in complex media (Bidochka et al. [1987]; Chong-Rodríguez et al. [2011]; Hegedus et al. [1990]; Humphreys et al. [1990]; Pham et al. [2009]; Rombach [1989]; Safavi et al. [2007]; Samsináková [1966]; Thomas et al. [1987]; Vega et al. [2003]). However, only a few publications deal with the production of mycelium (Núñez-Ramírez et al. [2012]) and the production of BS in complex (Humphreys et al. [1989,1990]) and mineral media (Lane et al. [1991]) by submerged fermentation, respectively. Endophytic *B. bassiana* strains can exist asymptomatically in a variety of plants like banana (Akello et al. [2008]), opium poppies (Quesada-Moraga et al. [2006]), maize (Bing and Lewis [1992]) and sorghum (Tefera and Vidal [2009]). The recently isolated endophytic *B. bassiana* strain ATP-02 showed great potential for a novel plant control measure in a variety of crops (Tefera and Vidal [2009]). However, it remained unknown if this strain can be mass-produced to high yields and if the spores from a submerged fermentation are able to colonize plants. That is why the objective of the present work was to produce spores of endophytic *B. bassiana* ATP-02 in a cost-effective culture medium on lab-scale and to scale-up the process to a 2 L stirred-tank reactor. Finally the virulence of the produced spores was checked in a bioassay with *Plutella xylostella* and their potential to colonize oilseed rape leaves via a leaf application was investigated.

## Materials and methods

All materials used were purchased from Merck KGaA (Darmstadt, Germany), Carl Roth GmbH (Karlsruhe, Germany) or AppliChem GmbH (Darmstadt, Germany), if not mentioned otherwise. Sugar beet molasses with a dry matter content of 80% consisting of 50% sucrose was purchased from Suedzucker AG (Mannheim, Germany). All concentrations are given as (w/w).

### Strain

*B. bassiana* isolate ATP-02, DSM 24665, was provided by Prof. Stefan Vidal, Georg-August-University, Department of Crop Sciences/Agricultural Entomology, Goettingen, Germany. The strain was raised at 25°C on SDA agar containing 1% casein peptone, 2% glucose and 1.5% agar-agar at pH 5.5. Temperature optimum was found at 25°C and pH optimum at 5.5 (data not shown).

### Cultivation in shake flask culture

Different liquid media were used to cultivate *B. bassiana*: TKI medium with 5% carbon source (Thomas et al. [1987]), Czapek-Dox medium (Kučera [1971]), YPG medium and PG medium (Bidochka et al. [1987]), Vogel’s medium (Vogel [1956]), SD medium (Odds [1991]), CGM medium containing 1% glucose, 1% corn steep liquor, 0.5% NaCl, 0.1% NaNO_3_, 1% CaCO_3_ (Samsináková [1966]), PWG medium containing 1% glucose, 8.75% whey powder, 0.25% peptone (Kassa et al. [2008]), YG medium containing 1% glucose, 1% yeast extract (Leckie et al. [2008]), YSM medium containing 2% sucrose, 0.5% yeast extract, 0.15% KH_2_PO_4_, 0.05% MgSO_4_∙7H_2_O, 0.001% CaCl_2_, 0.000003% H_3_BO_3_, 0.000004% MnSO_4_∙4H_2_O, 0.0000025% Na_2_MoO_4_∙2H_2_O, 0.000008% CuSO_4_∙5H_2_O, 0.00004% ZnSO_4_∙7H_2_O, 0.00005% FeCl_3_∙6H_2_O, 0.00004% CoCl_2_∙6H_2_O (Rombach 1988, 1989) and YS medium containing 2.5% sucrose, 2.5% yeast extract (Rombach [1989]). In each case 50 mL medium was placed in 250 mL DURAN® baffled flasks. The pH values of the media were adjusted to 5.5 with 0.5 M NaOH. As a starter inoculum AC from SDA agar (see above) were used. The AC were isolated by flooding the plates with 2 × 5 mL of sterile 0.1% Tween 80 and gently raking the plates with a sterile bristle brush. The shake flask cultures were inoculated with the spore suspension to give an initial spore density of 5.0×10^4^ AC/mL. The flasks were incubated at 25°C on a rotary shaker at a speed of 150 rpm for 8–10 days. Every day, 1 mL samples were taken to check developmental stage and the concentration of the spores with a Thoma counting cell chamber under 400 × magnification (photomicroscope, Carl Zeiss AG, Oberkochen, Germany).

### Fermentation

Batch fermentation was carried out in a 2 L BIOSTAT® Bplus stirred tank reactor (Sartorius Stedim System GmbH, Guxhagen, Germany) with a working volume of 1.5 L. The basal salts were dissolved in 1200 mL ddH_2_O and were autoclaved in the bioreactor. Also, a few drops of the anti-foam agent Pluronic® PE 8100 (BASF SE, Ludwigshafen, Germany) were added before fermentation started. Likewise, 300 mL of a carbon source stock solution (75 g carbon source) were autoclaved separately and were inoculated with 7.5×10^8^ aerial conidia (5.0×10^4^ spores/mL). To start the fermentation, the inoculum suspension was added to the bioreactor. Temperature was maintained at 25°C and fermentation time was between 8–10 days.

### Analysis

The metabolic respiratory quotient (RQ) is an on-line parameter for the formation of biomass was calculated from the ratio of the generated carbon dioxide and the consumed oxygen, which were measured with an O_2_ and CO_2_ sensor (BlueSens GmbH, Herten, Germany) in the exhaust air. For the determination of fungal dry biomass 15 mL samples were centrifuged for 10 min at 20,000 *g*, washed two times with ddH_2_O and centrifuged again. The pellets were suspended in 5–7 mL of ddH_2_O. The cell suspensions were dried at 115°C to constant weight using a moisture analyzer (Sartorius AG, Goettingen, Germany). Each time, determination of fungal dry biomass was carried out in two replicates.

The colony forming units (CFU) of BS and SCS were determined by spreading 100 μL of diluted samples on SDA plates (Odds [1991]) and incubating at 25°C for 4–6 days. To ensure that a sample will yield CFU in a range between 50 and 150 colonies requires several 10-fold dilutions of the sample with 0.9% NaCl. The CFU were determined on duplicate samples. The CFU/ml was calculated as follows:

(1) $$\frac{\text{CFU}}{\text{mL}}=\frac{\text{CFU}}{\text{plate}}\cdot \frac{\text{dilution}\;\text{factor}}{0.1\;\text{mL}}$$

### Insect virulence assay

Bioassays were conducted with 30 second instar larvae of *Plutella xylostella* L. (Yponomeutidae: Lepidoptera), which were provided by Prof. Stefan Vidal, Georg-August-University, Department of Crop Sciences/ Agricultural Entomology, Goettingen, Germany. The culture broth of the fermentation was centrifuged for 5 min at 20,000 *g*, washed two times with ddH_2_O and centrifuged again. The washed spore mix, consisting of 95% BS and 5% SCS, as well as pure AC from a two-weeks-old SDA culture were suspended in 0.1% Triton-X114 to obtain a final concentration of 10^6^ viable spores/mL. Aliquots of 1 mL of the suspensions were brushed on the adaxial side of secondary oilseed rape leaves with an area of 80 ± 10 cm^2^. The control leaves were treated with 0.1% Triton-X114 only. High 500 mL beakers were filled with 100 mL water agar (1.0% agar-agar). In each case three stalks of the treated leaves were drilled into the solid water agar. The upper surface of the water agar was covered with sterile filter paper to prevent the larvae from getting stuck. Afterwards, ten larvae were transferred into each of the beakers with and without spores, respectively. The beakers were closed with silk gauze and incubated at room temperature.

After 14 days, the dead larvae were surface sterilized with 70% ethanol for 2 min, 5% sodium hypochlorite for 3 min and 70% ethanol for 2 min, rinsed twice in sterile distilled water, and then placed on sterile tissue paper in a laminar airflow cabinet. The larvae were placed on a modified *B. bassiana* selective medium, consisting of 1% casein peptone, 4% glucose, 0.1375% Syllit® (Spiess-Urania Chemicals GmbH, Hamburg, Germany), 0.0005% chlortetracycline, 0.0005% crystal violet and 1.5% agar-agar (Chase et al. [1986]; Rangel et al. [2010]), and were incubated at 25°C for 1 week. To evaluate the efficacy of the surface sterilization method the water used to rinse the tissues after surface sterilization was plated on selective medium and was incubated, too.

After re-isolation, the DNA of the mycelium was extracted. In each case 50 mg mycelium were cooled on ice, and 1 mL CTAB buffer (0.02 M Na-EDTA, 126 mM sorbitol, 36.8 mM n-laurylsarcosine, 22 mM CTAB, 90 mM polyvinylpyrrolidone, 10 mM Tris, 0.8 M NaCl at pH 8.0), 2 μL β-mercaptoethanol and 1 μL proteinase K (0.1 g/L) were added. The samples were incubated at 42°C for 10 min, at 65°C for 10 min, and then 800 μL chloroform:isoamylalcohol 24:1 were added, mixed, stored on ice for 10 min and centrifuged at 8,000 rpm for 10 min. The supernatants were carefully taken, mixed with 100 μL 5 M NaCl and 200 μL 30% PEG, stored for 10 min at room temperature and centrifuged at 14,000 rpm for 15 min. The pellets were washed twice with 600 μL 75% ethanol and dried in a thermoblock at 65°C. Afterwards, the DNA pellet was dissolved in 100 μL TE buffer (10 mM Tris at pH 8.0) and stored at −20°C. All samples were assessed by PCR using specific primers namely RD1-F (5′- TGGGTATAGGCCGCAGCAC-3′) and RD1-R (5′- CTCTAAGGGTGACAGGGATAG-3′) which amplify a 208-bp region of the ITS1-5.8S-ITS2 region of *B. bassiana.* The primer sequences were compared with the NCBI nucleotide database using BLAST and were predicted to be 100% homologous to *B. bassiana* isolate DAOM210087 as well as ATP-02. All amplifications were performed in a TProfessional (Biometra GmbH, Goettingen, Germany) thermocycler. PCR reactions consisted of a reaction mix (final volume 10 μL) of 1 μl 10 x reaction buffer, 2.5 mM MgCl_2_, 100 μM dNTPs, 0.3 μM RD1-F primer, 0.3 μM RD1-R primer, 0.3 U Taq polymerase (5 Prime GmbH, Hilden, Germany) and 1 μl template DNA (30 ng). The cycling program included an initial denaturation step of 5 min at 95°C, followed by 35 cycles of 1 min denaturation at 95°C, 1 min annealing at 59°C, and 1 min extension at 72°C. Amplification products were mixed with GelRed™ (Biotium Inc., Hayward, Canada), separated by electrophoresis in 1.5% agarose gels in 1× TBE buffer (89 mM Tris, 89 mM boric acid, 2 mM EDTA at pH 8.0) for 60 min at 100 V and visualized under UV radiation.

All tests were run for 14 days and each test was repeated thrice. The mortality data were analyzed statistically using one-way ANOVA test.

### Penetration assay

Native oilseed rape (*Brassica napus* L.) cultivar “PULSAR” was obtained from the Deutsche Saatveredelung AG (Lippstadt, Germany). The seeds were planted singly in pots containing sterile soil/sand substrate (1:3 (v/v)) (Fruhstorfer Erde Type T25, HAWITA Group GmbH, Vechta, Germany) and were cultivated under greenhouse conditions, The plants were maintained at 18-22°C, 40–60% RH for 16 weeks and with a 12-h photoperiod (SON-T Agro 400 W, Philips, Amsterdam, Netherlands).

Formulation components consisting of 0.1% Triton X-114 as a wetter, 0.1% gelatine 280 Bloom (Gelita AG, Goeppingen, Germany) as a humectant, 1% sugar beet molasses as nutrient and 1% titanium dioxide as a UV protection agent were autoclaved for 20 min at 121°C. The spore suspension from a submerged fermentation was centrifuged for 5 min at 20,000 *g*, washed twice with ddH_2_O and centrifuged again. Afterwards the spores were suspended in 0.9% NaCl and added to the formulation components up to a final concentration of 10^6^ spores/mL. The control formulation was free of fungal biomass. Then, the formulations were brushed onto an area of approximately 3 cm of the tips of 9^th^ secondary leaves oilseed rape plants. To increase the relative humidity up to 95%, the treated leaves were wrapped with plastic bags for the first 48 h. After 7 days the leaf tips were cut off and the untreated base of the leaves were harvested for the detection of endophytic colonization with *B. bassiana* by microscopy and PCR.

For microscopy, cross-sections of the leaf mid rip were stained with 0.5% rose bengal dissolved in 5% aqueous ethanol for 15 sec and were washed with ddH_2_O (Saha et al. [1988]). Growth of *B. bassiana* in the plant tissue was detected at 200-fold magnification with a light microscope. Afterwards, the leaves were surface sterilized as mentioned above and then placed on sterile tissue paper in a laminar airflow cabinet. In a preliminary test it was shown that this surface sterilization method kills all spores which were applied onto oilseed rape leaves.

For DNA extraction which was described above, approximately 400 mg plant tissue from the surface sterilized leaves and stems was taken. The plant tissue was crushed in a MM400 ball mill using a sterile 5 mm steel ball (Retsch GmbH, Haan, Germany) for 5 min at 30 Hz. To isolate *B. bassiana* DNA from a SDA culture, 50 mg fungal biomass were directly used for DNA extraction. All tissue samples of both treated and untreated oilseed rape plants were assessed by PCR.

## Results

### Screening of media in shake flask culture

**Figure 1:**
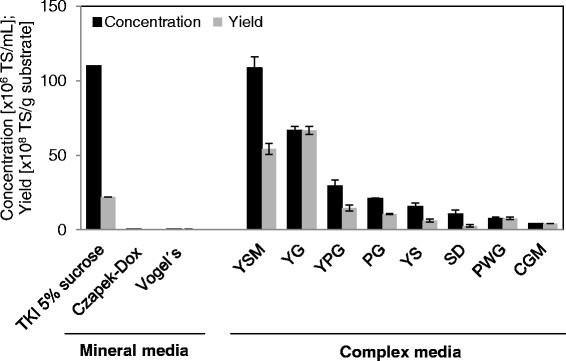
**Influence of different culture media on spore formation.***B. bassiana* was cultivated in 250 mL shake flasks (n = 2). Mean (±SD) concentrations and mean (±SD) yields of TS 168 h after inoculation.

The entomopathogenic and endophytic fungus *B. bassiana* isolate ATP-02 was cultivated in shake flasks. The different liquid media described above were used to study the effect of various nutrients, basal salts and other complex components on submerged spore formation. In Figure 1 the concentrations and yields of total spores (TS) with regard to the different culture media are illustrated. The most promising culture medium with regard to a maximum growth and optimum sporulation was the TKI medium with 5% sucrose as a carbon source: After a cultivation time of 168 h *B. bassiana* produced TS in a concentration of 1.10 ± 0.01×10^8^ TS/mL consisting of 100% BS. In this TKI medium a yield of 2.20 ± 0.02×10^9^ TS/g sucrose was obtained. Due to the lower substrate concentration of 2% sucrose and the also high concentration of 1.09 ± 0.08×10^8^ TS/mL in the YSM medium a yield of 5.43 ± 0.38×10^9^ TS/g sucrose was obtained. However, in contrast to the TKI medium, the YSM medium contains 0.5% yeast extract as a complex component. In all cases, the achieved concentration of SCS was lower than 11% of the TS. The highest, but still low SCS concentration of 0.29 ± 0.04×10^6^ SCS/mL was obtained in the YG medium.

### Influence of different carbon sources on spore formation of *B. bassiana*

**Figure 2:**
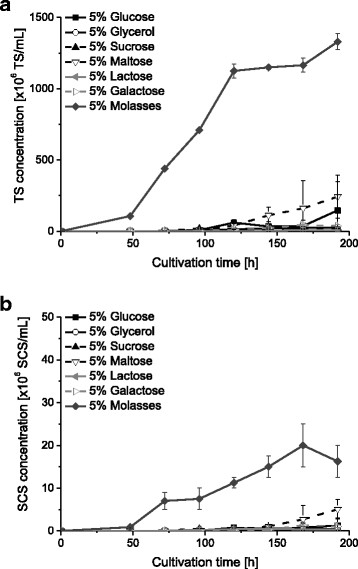
**Influence of different carbon sources of the TKI medium on spore formation.***B. bassiana* was cultivated in 250 mL shake flasks (n = 2). **(a)** Mean (±SD) concentration of BS. **(b)** Mean (±SD) concentration of SCS.

*B. bassiana* ATP-02 was cultured in TKI medium which was supplemented with 5% of different pure carbon sources according to Thomas et al. ([1987]). Furthermore, TKI medium was supplemented with 5% sugar beet molasses as a complex carbon source which consisted of 50% sucrose according to manufacturer specification (Suedzucker AG, Mannheim, Germany). In Figure 2 a and b the concentrations of TS and SCS with regard to the different carbon sources are illustrated. The highest concentration of 1.17 ± 0.05×10^9^ TS/mL corresponding to the highest yield of 4.68 ± 0.20×10^10^ TS/g sucrose was obtained in the TKI medium with 5% sugar beet molasses. Furthermore, in this medium *B. bassiana* also produced the highest concentration of 2.00 ± 0.50×10^7^ SCS/mL corresponding to a SCS yield of 8.00 ± 2.00×10^8^ SCS/mL 168 h after inoculation. However, the biomass consisted of more than 98% BS. In spite of the lower sugar concentration of the molasses (2.5%) the concentration of TS could be increased up to 11-times in contrast to the cultivation with 5% sucrose. Due to the lower sugar concentration the yield of TS could be even increased up to 21-times.

Interestingly, when TKI basal salts were omitted from the 5% molasses medium, TS concentration decreased by 96% compared to cultivation in the original TKI medium (data not shown).

### Fermentation of *B. bassiana* ATP-02

*B. bassiana* ATP-02 was raised in a 2 L stirred tank reactor to produce high concentrations of TS. Based on the cultivations in shake flasks *B. bassiana* ATP-02 was cultivated in the mineral TKI medium with 5% sugar beet molasses and were inoculated with 5×10^4^ AC/mL. The fermentation conditions and growth parameters are given in Table 1. During 48 h fermentation time, 12.6 g/L dry biomass was produced. Maximal specific growth rate μ_max_ was 0.14 h^−1^. Average doubling time was 14.7 h. The minimal doubling time of 4.9 h was reached between 48 and 72 h after inoculation. The results of the fermentation could be verified in 7 sequentially performed runs, which are shown in Table 2.

**Table 1:** **Cultivation of** ***B. bassiana*** **ATP-02 in a 2 L stirred tank reactor: fermentation conditions and growth parameters**

**Fermentation conditions**	
Inoculum [aerial conidia/mL]	5×10^4^
Start pH [−]	5.5
Stirrer speed [rpm]	600
Aeration rate [vvm]	1.0
Aeration rate [L/min]	1.5
Temperature [°C]	25
**Growth parameters**	
Biomass produced (after 48 h) [g/L]	12.6
Max. specific growth rate μ_max_ [h^−1^]	0.14
Min. doubling time [h]	4.9
Mean doubling time [h]	14.7

**Table 2:** **Cultivation of** ***B. bassiana*** **ATP-02 in 7 sequentially performed fermentation runs**

**No.**	**Total spores [x10**^**9**^** TS/mL]**	**Submerged conidiospores [x10**^**9**^**SCS/mL]**
1	1.11 ± 0.00	0.04 ± 0.00
2	1.45 ± 0.01	0.10 ± 0.01
3	1.12 ± 0.01	0.06 ± 0.01
4	2.08 ± 0.02	0.12 ± 0.01
5	1.89 ± 0.01	0.09 ± 0.02
6	1.39 ± 0.04	0.08 ± 0.01
7	1.11 ± 0.09	0.04 ± 0.01

Achieved mean (±SD) concentrations of TS and SCS 168 h after inoculation. Standard deviations were calculated from two technical replicates.

**Figure 3:**
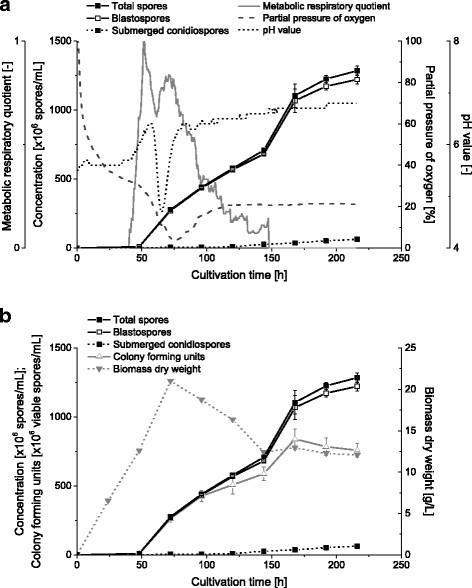
**Cultivation of*B. bassiana*in a 2 L stirred tank reactor.** The figure shows the mean (±SD) concentrations of TS, BS and SCS. **(a)** Process parameters. **(b)** Correlation of spore counts with biomass and mean (±SD) CFU. In each case, standard deviations resulted from two technical replicates.

The Figure 3a and b show the details of the fermentation no. 1. The fermentation process can be subdivided in two phases: a phase of mycelium formation and a following phase of spore formation.

At the beginning of the fermentation the amount of dry biomass increased because the fungus produced mycelium. After 62 h a sudden decrease of the RQ to 0.6 followed by a sharp decrease of pH to 4.7, a short recuperation of RQ and a decrease of pO_2_ to 4% was observed. A sample taken shortly thereafter at 72 h still yielded 21 g biomass/L and the broth was still visibly viscous. Then, biomass dry weight decreased which was accompanied by a visible reduction of mycelium. Preliminary HPLC data indicated a formation of oxalate (data not shown). At this time the concentration of TS started to increase up to 1.29 ± 0.04×10^9^ TS/mL corresponding to a yield of 5.16 ± 0.16×10^10^ TS/g sucrose at the end of the fermentation. However, the biomass consisted of more than 95% BS. 96 h after inoculation the viability of TS started to decrease. The maximum concentration of 0.84×10^9^ viable spores/mL corresponding to a yield of 3.36×10^10^ viable spores/g sucrose was obtained 168 h after inoculation. During the further fermentation process the concentration of viable spores decreased to 0.78×10^9^ TS/mL corresponding to a yield of 3.12×10^10^ TS/g sucrose at the end of the fermentation. Besides, the biomass dry weight decreased during the spore formation phase from 21 g/L to 12 g/L. Furthermore, 80 h after inoculation the RQ decreased continuously and the pO_2_ reached a non-critical value of 20% and a clogging of the pO_2_ electrode was noted.

### Insect virulence assay

**Figure 4:**
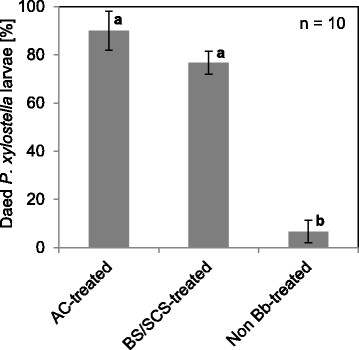
**Virulence test with*P. xylostella*larvae.** The larvae were fed with AC-treated as well as BS/SCS-treated (95% BS and 5% SCS) and non *B. bassiana* (*Bb*)-treated oilseed rape leaves. Means (±SD) followed by different letters are significantly different at P < 0.01 using one-way ANOVA test. In each case, standard deviations resulted from three replicates with 10 larvae.

The spore mixture from a submerged fermentation, consisting of 95% BS and 5% SCS as well as pure AC harvested from a petri dish were applied in a virulence test against the diamondback moth, *P. xylostella*. After 14 days, in the control without fungal spores 93 ± 5% of the larvae developed into viable adult insects. However, 77 ± 5% of larvae fed with spore mix-treated leaves as well as 90 ± 8% of larvae fed with AC-treated leaves died within a week (Figure 4). The dead larvae were surface sterilized, placed on a *B. bassiana* selective medium and mycelium grew out of all larvae treated with fungal spores and the mycelium was identified as *B. bassiana* by PCR. The mycelium which grew out of dead larvae not treated with *B. bassiana* was clearly not *B. bassiana*, so that these larvae did not die by *B. bassiana* induced mycosis. It was observed that the spores from submerged fermentation (P < 0.01; F_1,4_ = 220.5) as well as the pure AC (P < 0.01; F_1,4_ = 156.3) significantly affected the mortality of larvae. Besides, the number of dead larvae was not significantly affected by the type of spores.

### Penetration assay

**Figure 5:**
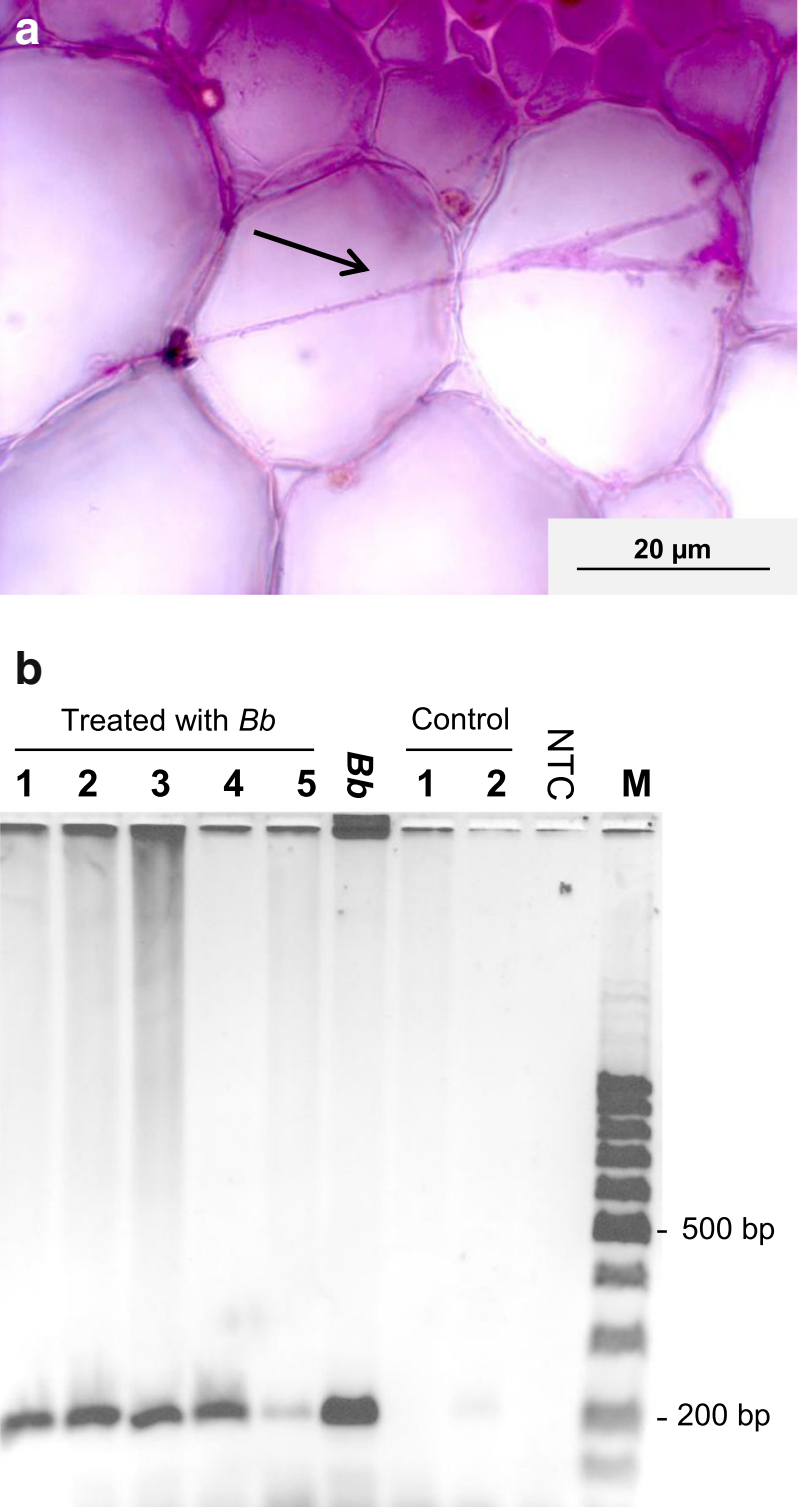
**Penetration assay with*B. bassiana*ATP-02.** Spray formulations with (n = 7) and without (n = 2) *B. bassiana* (*Bb*) were brushed onto the leaf tips. **(a)** Microscopic detection of *Bb* in a cross-section of a leaf mid rip from a *Bb*-treated leaf at 200-fold magnification. The arrow marks the hyphae in the leaf tissue. **(b)***Bb* was detected in the untreated area of the *Bb*-treated leaves by PCR and subsequent gel electrophoresis. Lane 1–5: DNA from *Bb*-treated leaves; Lane 6: *Bb* DNA from pure SDA culture; Lane 7–8: DNA from control-treated leaves; Lane 9: no template control; Lane 10: 100 bp DNA ladder (AppliChem GmbH, Darmstadt, Germany).

The influence of a formulation with *B. bassiana* spores (10^6^ TS/mL) on the penetration of oilseed rape leaves was investigated. The formulation was brushed onto the 9^th^ secondary leaf tips of seven oilseed rape plants and afterwards, endophytic *B. bassiana* was detected in the tissue of the untreated leaf base by PCR and microscopy. After 7 days, no hyphae growth was observed microscopically in control leaves treated without *B. bassiana* (n = 2). However, hyphae growth was observed in the mid rip cross-sections of 100% of leaves treated with the formulation. A randomly selected cross-section of these leaf mid rips is illustrated in Figure 5a. To verify that the microscopically detected mycelium was *B. bassiana*, a PCR was performed. *B. bassiana* was detected in all untreated areas of the leaves treated with the formulation by PCR and subsequent gel electrophoresis. The positive PCR signals of five randomly selected leaves are shown in Figure 5b and no PCR amplification was observed in control plants.

## Discussion

This study deals with the fermentation aspects of the endophytic *B. bassiana* ATP-02 which might prepare the way to exploit endophytes as commercial biocontrol agents.

### Screening of media in shake flask culture

In the past, mass-production of *B. bassiana* has focused on AC, but the production through surface cultivation or a two-stage process in which the fungus is allowed to develop under submerged conditions and subsequently transferred to a solid media to sporulate requires long cultivation times, large amounts of space and can be labour-intensive (Hall and Papierok [1982]). The obvious advantages of a submerged cultivation are that the fungus produces spores in a relatively short time with high yields under controlled sterile conditions as well as a simpler scale-up in contrast to solid-state fermentation (Feng et al. [1994]; Hegedus et al. [1992]; Patel et al. [2011]). It was previously known that *B. bassiana* strains grow in a variety of liquid mineral and complex media, but that the conidiation of the fungi under submerged conditions may be strain-specific (Kassa et al. [2008]) and needs to be investigated for each strain in detail. Furthermore, it was hypothesized that the endophytic *B. bassiana* strain ATP-02 does not grow in the same way as the established *B. bassiana* strains that are applied in classic biocontrol on the surface of plants or in the soil.

However, results on cultivation in shake flasks showed that *B. bassiana* ATP-02 was able to produce BS in the described culture media. Generally, in a submerged cultivation *B. bassiana* can produce two types of spores, namely BS and SCS. BS are relatively large, thin-walled and single-celled hyphal bodies (Bidochka et al. [1987]). SCS, on the other hand, are small, spherical, more uniform in size and show a higher shelf life than BS (Thomas et al. [1987]; Hegedus et al. [1992]; Holder et al. [2007]). They arise from the fungal mycelia or directly from BS in a process known as microcycle conidiation (Smith et al. [1981]). Thomas et al. ([1987]) describe the direct formation of SCS from BS in the mineral TKI medium with 5% glucose after a cultivation time of 96 h. This phenomenon was not observed in the present work. From the biotechnological point of view the most important properties of the culture medium are high yields of TS and SCS, respectively. Although a higher TS yield was obtained in the YSM medium consisting of basal salts, 2% sucrose and 0.5% yeast extract a 2.5-fold, the mineral TKI medium without expensive complex components was further optimized because of cost-effectiveness.

### Influence of different carbon sources on spore formation of *B. bassiana*

The highest concentrations and yields of TS and SCS were obtained in the TKI medium with 5% sugar beet molasses as a carbon source. It should be pointed out that the concentration of TS could be increased up to 11-times in contrast to the cultivation with 5% sucrose in spite of the lower sucrose concentration of 2.5% in the molasses. The utilized sugar beet molasses consisted of 50% sucrose and only traces of other sugars like glucose, fructose and raffinose as well as different proteins and basal salts according to manufacturer specification (Suedzucker AG, Mannheim, Germany). Furthermore, it could be shown that in addition to the present basal salts of the sugar beet molasses the TKI medium is necessary for optimal growth of *B. bassiana*. Sugar beet molasses is a residue of the agricultural industry and consequently, it is a low-cost source, which is a big advantage compared to other carbon sources. Therefore, the cost of 1 L TKI basal medium amended with 5% sugar beet molasses amounts to only 0.31 €.

### Fermentation of *B. bassiana* ATP-02

After the optimized cultivation of *B. bassiana* ATP-02 in shake flasks the process was scaled-up to a 2 L stirred tank reactor. Based on the cultivations in shake flasks *B. bassiana* ATP-02 was cultivated in the TKI mineral medium with 5% sugar beet molasses. In a preliminary test the fermentation was inoculated with a 5-days old shake flask culture of *B. bassiana*, which contained only BS. During the fermentation the fungus produced only mycelium (data not shown). Since the objective of cultivation was a high concentration of TS, further fermentations were inoculated with 5.0×10^4^ AC/mL. The achieved concentrations and yields of TS and SCS were comparable with the cultivation of *B. bassiana* ATP-02 in shake flasks and could be verified in 7 sequentially performed fermentation runs.

An unusual point during all fermentations is the sudden decrease of biomass dry weight in correlation with the low concentration of oxygen in the culture broth 72 h after inoculation. At this point it was observed that the finely dispersed mycelium lysed and the pH and the viscosity of the culture broth decreased. HPLC analysis indicated presence of oxalate. The reason for the rapid pH decrease in our fermentation is not clear, but it can be presumed that intracellular oxalate was suddenly released due to the lysis of mycelium. The following increase of pH suggests that oxalate was then metabolized by the growing spores. These presumptions are supported by other studies which also indicate that *B. bassiana* strains are able to produce, secrete and metabolize oxalate in vitro (Bidochka and Khachatourians [1993]; Kirkland et al. [2005]).

During the fermentation process the pH value was not regulated due to the fact that fluctuating pH values between 4.0 and 6.5 have no considerable impact on the growth of *B. bassiana* (Padmavathi et al. [2003]; Thomas et al. [1987]). The typical limitation of oxygen can be prevented by increase of the stirrer speed or agitation rate (Patel et al. [2011]). But the primary objective of this fermentation process was not the production of finely dispersed mycelium but rather the mass-production of sprayable TS without any mycelium.

The recurring decrease of biomass dry weight in the spore formation phase cannot be explained in detail. It may be hypothesized that the mycelium is decreasing but the spore formation does not compensate the weight loss. It can be ruled out that spore biomass was lost during sample preparation as biomass was not filtered but centrifuged.

Furthermore, the achieved TS concentration and yield of the described fermentation process was higher than those obtained by other investigators in studies of liquid shake flask cultivations of epiphytic *B. bassiana* strains. For example, Thomas et al. ([1987]) reported a maximum concentration of 5.0×10^8^ TS/mL corresponding to a yield of 1.00×10^10^ TS/g glucose, Rombach ([1989]) described that *B. bassiana* produced TS in a maximum concentration of 0.17×10^9^ TS/mL corresponding to a yield of 0.85×10^10^ TS/g sucrose, Vega et al. ([2003]) obtained a maximum concentration of 1.24×10^9^ BS/mL corresponding to a yield of 1.65×10^10^ BS/g glucose and Pham et al. ([2009]) reported a maximum concentration of 0.85×10^9^ BS/mL. The highest described concentration of BS was reported by Chong-Rodríguez et al. ([2011]), who obtained an inconsistent concentration of 6.38×10^9^ ± 3.63×10^9^ BS/ml in a somewhat costly complex medium, which consisted of 5% sucrose, 2% corn steep liquor and basal salts. In comparison to other published data on cultivation of *B. bassiana* isolates in a solid-state or submerged cultivation it can be shown that the described fermentation process is very economical with regard to the achieved concentration and yield of TS. Furthermore, an obvious advantage of the fermentation process is that the cost of 10^12^ TS amounts to only 0.24 € with regard to the utilized culture medium. A further increase of the TS concentration can likely be realized by fed-batch fermentation.

### Insect virulence assay

The mortality was 77 ± 5% for *P. xylostella* larvae, which were fed with oilseed rape leaves treated with BS and SCS, and 90 ± 5% for larvae fed with AC-treated leaves. These larvae mortalities are in accordance with those obtained by other investigators. Godonou et al. ([2009]) reported that AC of *B. bassiana* caused *P. xylostella* larvae mortality ranging from 20 to 94%. BS which were sprayed on *P. xylostella* larvae showed a mortality ranging from 95 to 100% (Fargues et al. [1983]). In addition, Chong-Rodríguez et al. ([2011]) described that BS of *B. bassiana* maintained for six months at 4°C showed a mortality of more than 80% against third-instar *P. xylostella* larvae 8 days after application. Furthermore, Ortiz-Urquiza et al. [2010] observed that the composition of the culture medium affected the virulence of AC from *B. bassiana* because of an increased or decreased secretion of virulent proteins. But the influence of the culture media on the virulence of *B. bassiana* was not investigated in this work. Since no further mortality tests were conducted with pure BS and pure SCS, it can only be hypothesized that BS must have killed the larvae, because the spore mix consisted of 95% BS which are the preferred propagule of *B. bassiana* in the haemocoel of infected insects (Jackson et al. [2010]; Shimizu et al. [1993]; Sieglaff et al. [1997]). Furthermore, BS are highly infective against a number of insect pests and have a lower LD_50_ when compared to AC or SCS (Hegedus et al. [1992]). Finally, it was shown here that spores from a submerged cultivation are as virulent to *P. xylostella* larvae as AC.

### Penetration assay

The simple penetration assay indicated that the spores from submerged fermentation show endophytic properties. This is in line with studies on AC that were applied to leaves and could to some extent colonize plants (Bing and Lewis [1991,1992]; Gurulingappa et al. [2010]; Landa et al. [2013]; Posada et al. [2007]; Quesada-Moraga et al. [2006]; Quesada-Moraga et al. [2009]; Tefera and Vidal [2009]; Wagner and Lewis [2000]). Many questions about endophytism remain that are not within the scope of this fermentation study.

To the best of our knowledge, this is the first report of fermentation of an endophytic *B. bassiana* strain in a low-cost culture medium to very high yields of TS, which are able to penetrate oilseed rape leaves via a leaf application. This should further encourage the recent activities to exploit the biocontrol potential of endophytic entomopathogenic fungi. Besides, the evidence that the endophytic strain grows in simple cultivation conditions much like the classic biocontrol strains is further proof that in nature some microorganisms are facultative endophytes, which can optionally live inside plants and other habitats (Hardoim et al. [2008]). The results also clearly suggest to further explore submerged cultivation for entomopathogenic fungi in general. Further studies are required to produce higher amounts of SCS of *B. bassiana*, which show a higher shelf life than thin-walled BS and may also persist longer when sprayed onto plant leaves.

## Abbreviations

AC: Aerial conidia: 

BS: Blastospores: 

SCS: Submerged conidiospores: 

TS: Total spores: 

RQ: Respiratory quotient: 

CFU: Colony forming units: 

## Competing interest

The authors declare that they have no competing interests.

## Authors’ contributions

RL designed and carried out the cultivations, fermentations and mortality tests, analyzed the data and wrote the manuscript. DJS carried out the molecular biology studies. AVP conceived of the study, and participated in its design and coordination and helped to draft the manuscript. All authors read and approved the final manuscript.
